# The effectiveness of controlled ovarian stimulation with tamoxifen for patients with estrogen‐sensitive breast cancer: A systematic review and meta‐analysis

**DOI:** 10.1002/rmb2.12543

**Published:** 2023-09-23

**Authors:** Tsukasa Yoshida, Osamu Takahashi, Yoko Suzuki, Erika Ota, Tetsuya Hirata

**Affiliations:** ^1^ Department of Obstetrics and Gynecology St. Luke's International Hospital Tokyo Japan; ^2^ St. Luke's International University Tokyo Japan

**Keywords:** aromatase inhibitors, breast neoplasms, fertility preservation, reproductive techniques, tamoxifen

## Abstract

**Purpose:**

Tamoxifen is used for the suppression of estrogen‐sensitive tumor recurrence in oocyte retrieval cycles. This meta‐analysis aimed to evaluate the quality of controlled ovarian stimulation (COS) with co‐administration of gonadotropins and tamoxifen (COS with tamoxifen).

**Methods:**

PubMed, Embase, and Cochrane Library were searched for articles on October 30, 2022. The authors included studies comparing COS with tamoxifen and COS with gonadotropins and letrozole (COS with letrozole) or gonadotropin only (COS with gonadotropin only) for fertility preservation in patients with breast cancer. The main outcome measures were the COS quality, total number of retrieved oocytes (TOR), total number of mature oocytes (TMO), and peak estradiol levels (PEL).

**Results:**

Four studies (348 patients, two randomized controlled trials, and two cohort studies) were included in our meta‐analysis. There was no significant difference in TOR (95% CI, [−3.84, 2.90]) and TMO (95% CI, [−2.20, 2.64]) between COS with tamoxifen and COS with letrozole. There was also no difference in TOR (95% CI, [−6.14, 1.86]) between COS with tamoxifen and COS with gonadotropin only. Statistically significant decrease was observed in PEL during COS with letrozole compared with tamoxifen (95% CI, [1414.4, 4953.7]).

**Conclusions:**

The quality did not differ between COS with tamoxifen and COS with letrozole or gonadotropin only.

## 1 INTRODUCTION

Estradiol levels during in vitro fertilization (IVF) oocyte retrieval cycles increase 2 to 5 times more than their levels during natural conception. These high levels of estradiol persist throughout the pregnancy. It has been hypothesized that elevated estradiol levels during controlled ovarian stimulation (COS) in IVF increase the rate of breast cancer (BC) recurrence, as high levels of estradiol promote BC cell proliferation and angiogenesis.

In 2003, Oktay et al. proposed a protocol for COS with the co‐administration of gonadotropins and a selective estrogen receptor modulator, tamoxifen, to block estrogen receptors in breast tissue (COS with tamoxifen).^1^ Subsequently, Meirow et al. published a study on the safety and efficiency of COS with tamoxifen.^2^ In 2006, Oktay et al. proposed a COS protocol with the co‐administration of gonadotropins and an aromatase inhibitor, letrozole to suppress estradiol elevation in patients with estrogen receptor‐positive BC (COS with letrozole).^3^ These protocols have been widely used as the main COS methods for patients with estrogen receptor (ER)‐positive BC worldwide.

Blockage of the estrogen receptor and reduction in estrogen levels are associated with the suppression of the recurrence of estrogen‐sensitive tumors. However, it is not yet clear whether the quality of COS is affected by the use of tamoxifen and letrozole, in terms of the number of oocytes retrieved and the degree of maturation of oocytes.^4^, ^5^

The last systematic review that compared tamoxifen and letrozole for COS was published in 2013 from Cochrane review. The review concluded that it was difficult to determine the effectiveness of protocols using aromatase inhibitors due to the lack of high‐quality randomized control studies.^6^ In the last 10 years, various interventional and observational studies have been published worldwide. Yet, an updated systematic review and meta‐analysis has not been published. Therefore, whether COS with tamoxifen or COS with letrozole for BC patients still currently depends on regional practice patterns.

The objective of this systematic review and meta‐analysis was to evaluate the quality of COS (i.e., the number of oocytes retrieved and the oocyte maturation rate) with tamoxifen or letrozole in patients with ER‐positive BC. This review will be beneficial for the management of patients who require high‐quality fertility preservation techniques.

## 2 METHODS

### 2.1 Study design

This systematic review included individual and cluster randomized control trials (RCT), cohort studies, and case–control studies evaluating the quality of the COS (i.e., the number of oocytes retrieved and oocyte maturation rate) protocol involving the use of tamoxifen for patients with ER‐positive BC. We followed the Preferred Reporting Items for Systematic Reviews and Meta‐Analyses (PRISMA)^7^ and Meta‐analysis of Observational Studies in Epidemiology (MOOSE)^8^ protocols. The protocol for this review was registered in PROSPERO (registration number: CRD42023388846).

### 2.2 Search strategy

We searched the PubMed, Embase and the Cochrane Library databases. Additionally, other relevant studies were identified by searching the reference lists of pertinent articles. Unpublished studies were identified using the ClinicalTrials.gov registry (http://ClinicalTrials.gov/).

The electronic databases were searched from their inception until October 30, 2022. Key search terms were as follows: breast neoplasms [MeSH], aromatase inhibitor [MeSH], selective estrogen receptor modulator [TW] or tamoxifen [TW], fertility preservation [MeSH], etc. (details of search terms are included in Appendices 1, 2, and 3).

### 2.3 Eligibility criteria

Study design: individual and cluster RCTs, cohorts, and case–control studies.

Languages: Studies were not limited by their year of publication and language.

Exclusion criteria: Other observational studies (lowers/editorials, qualitative studies, therapy‐treatment studies, prognosis studies, cross‐sectional studies, case reports, case series, and gray literature) were excluded.

### 2.4 Population

Inclusion: Patients aged >18 years who underwent IVF for fertility preservation were eligible for inclusion in this study if they were diagnosed with BC. The patient inclusion criteria were not limited to those with ER‐positive breast cancer.

Exclusions: Patients under 18 years old and patients with other previous malignancies were excluded.

### 2.5 Intervention

Inclusion: Controlled ovarian stimulation with the co‐administration of gonadotropins and selective estrogen receptor modulator: tamoxifen (COS with tamoxifen).

### 2.6 Comparison

Inclusion: Control ovarian stimulation with the co‐administration of gonadotropins and the aromatase inhibitor: letrozole (COS with letrozole); Controlled ovarian stimulation with gonadotropins used only (COS with gonadotropin only).

### 2.7 Outcomes

#### 2.7.1 Primary outcomes

・Quality of COS, which was defined as total number of retrieved oocytes (TOR) and the total number of mature oocytes (TMO).

#### 2.7.2 Secondary outcomes

・Peak estradiol level (PEL) during the oocyte retrieval cycle: peak estradiol level was set as the secondary outcome as estrogen promotes the development and growth of BC cells through the alkylation of cellular molecules and the generation of DNA‐damaging active radicals, as previously demonstrated by an experimental study.^9^

### 2.8 Subgroup analysis

Subgroup analysis was also performed by pooling estimates for similar patient subsets among the studies, where available. The quality of COS was separately evaluated according to the type of COS, for example, antagonist and long gonadotropin‐releasing hormone (GnRH) agonist (long GnRH‐a).

### 2.9 Study selection and data extraction

Two independent investigators (T. Y. and Y. S.) searched the databases using a defined search strategy. Each search term has been presented in Supplementary Data. First, we selected studies by reviewing the titles and abstracts. They were then managed with Rayyan QCRI, which is a web application for systematic reviews. The individually recorded decisions of the investigators were compared. If there was a discrepancy, a third investigator (E. O) was consulted. After the titles and abstract section, each investigator evaluated the full text of the corresponding paper and determined whether it corresponded with the defined search strategy. Discrepancies were resolved by the third investigator. Finally, the selected papers were managed using the Paperpile application from Google.

After selecting the studies, two reviewers independently extracted data regarding the number of participants, number of cycles, patient characteristics, primary outcome measures, and secondary outcome measures. In case of missing data, the corresponding author was contacted.

### 2.10 Assessment of risk of bias

Two authors (T.Y. and Y.S.) independently assessed the Risk of Bias in the included RCTs using the Risk of Bias 2.0, (RoB 2.0) assessment tool outlined in the Cochrane Handbook for Systematic Reviews of Interventions.^10^ Six domains related to the risk of bias were assessed: domain 1, the randomization process; domain 2, deviations from the intended interventions; domain 3, missing outcome data; domain 4, measurement of the outcome; and domain 5, selection of the reported result. Authors reported their judgments as “low risk,” “some concerns” or “high risk” of bias. The outcomes reported in the protocol papers were compared, if available, to estimate domain 4. If not available, the corresponding authors of such studies were contacted or they were judged as “insufficient information.”

The Risk of Bias Assessment tool for Non‐Randomized Studies (RoBANS) was used to appraise the included non‐RCT studies.^11^ Six domains related to the risk of bias were assessed: selection of participants, selective outcome reporting, measurement of exposure, blinding of outcome assessments, incomplete outcome data, and selective outcome reporting. If the judgments of the two reviewers were inconsistent, we consulted a third reviewer (E.O.).

### 2.11 Statistical analysis

Two authors (T.Y. and Y.S.) independently analyzed the data using Review Manager version 5.4.1 (The Cochrane Collaboration, Oxford, England). The mean was estimated from the median, and standard deviations were calculated from the confidence interval (CI) limits, standard errors, or interquartile range, as required.

Dichotomous variables were summarized using the Mantel–Haenszel risk ratio or risk difference with 95% CI. Continuous variables were pooled and expressed as weighted mean differences (WMD) with a 95% CI. Heterogeneity across the studies was evaluated using *I* ^2^ statistics.^12^ Studies were considered not statistically homogeneous if *I* ^2^ < 50%. A random‐effects model was used as high heterogeneity was expected across studies. We assessed the evidence models for the outcomes of TOR, TMO, and PEL for the meta‐analysis using the GRADE system. As the number of eligible studies was less than ten, a funnel plot was not used to assess publication bias.

## 3 RESULTS

### 3.1 Study selection

**FIGURE 1:**
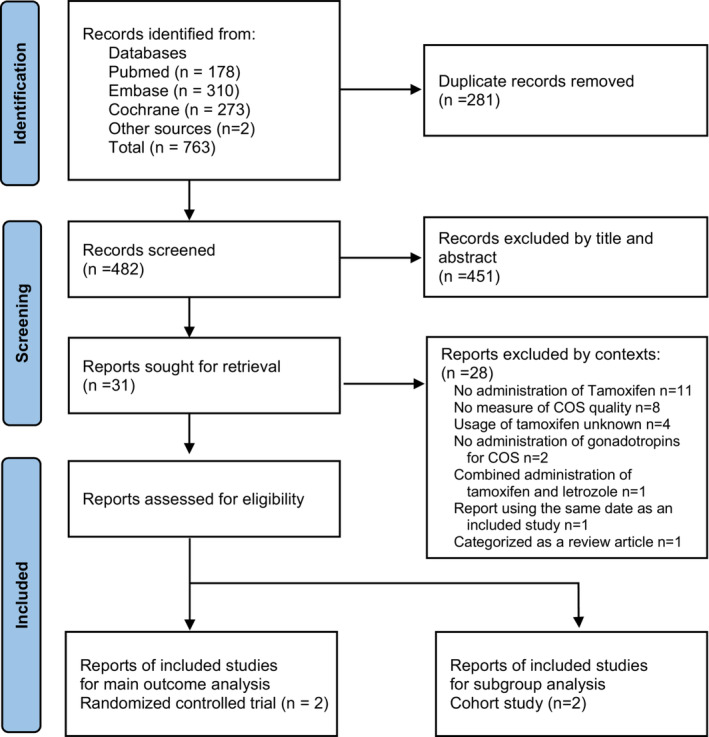
Preferred reporting items for systematic reviews chart; identification process for eligible studies.

A PRISMA flowchart has been demonstrated in Figure 1. We identified 763 studies from the databases and included one study from a different source. A total of 281 duplicate records were collected. The titles and abstracts of the remaining 482 articles were reviewed, 31 of which were eligible for full‐text evaluation. Finally, three studies (one RCT, one including both RCT and cohort studies, and one cohort study) were selected to match the predetermined criteria and were included in the analysis.

Two studies matched most of our selection criteria, but one study^5^ was excluded because the letrozole group, which was the control group, did not involve the use of gonadotropins for COS. In addition, one study^1^ was excluded because the control group without tamoxifen did not use gonadotropins for COS.

### 3.2 Study characteristics

Details of the selected studies have been demonstrated in Table 1, including study design, country, year, patient characteristics, total number of patients, mean age, and primary outcomes.

**TABLE 1:** Details of selected studies.

Study	Study design	Country	Duration	Numbers	Age (Mean ± SD) tamoxifen	Age (Mean ± SD) Letrozole	Age (Mean ± SD) gonadotropin	Measured outcome
Balkennende 2022	RCT	Netherlands	2014–2018	162	31.8 ± 4.4	32.3 ± 3.8	31.4 ± 4.0	TOR, TMO, PEL
Letoumeau 2021	RCT	USA	2016–2020	94	35.4 ± 5.0	33.7 ± 4.4	‐	TOR, TMO, PEL
Letoumeau 2021	Cohort	USA	2016–2020	82	35.4 ± 5.0	‐	32.5 ± 3.6	TOR, TMO, PEL
Meirow 2014	Cohort	Israel	N/A	70	34.1 ± 5.0	‐	33.0 ± 4.7	TOR, PEL

Abbreviations: PEL, Peak estradiol level; RCT, Randomized Controlled Trail; TMO, Total number of mature oocytes; TOR, Total number of oocytes retrieved; USA, United States of America.

Four studies (two RCTs and two cohort studies) included data from 408 patients with breast cancer. These studies were published in various countries (the United States, Europe, and the Middle East). Almost all studies reported TOR, TMO, and PEL; however, only one study^2^ did not report TMO.

### 3.3 Risk of bias of included studies

A summary of the risk of bias assessment for the two RCTs has been demonstrated in Table 2.^13^, ^14^

**TABLE 2:** Summary of Risk of Bias 2.0 assessment in all the Randomized control trials.

	Randomization process	Deviations from the intended interventions	Missing outcome data	Measurement of the outcome	Selective of the reported result	Overall
Balkennende 2022	Low	Low	Low	Low	Low	Low
Letoumeau 2021	Low	Some concerns	Low	Low	Low	Low
Low (%)	100	50	100	100	100	
Some concerns (%)	0	50	0	0	0	
High (%)	0	0	0	0	0	

The risk of bias in the randomization processes of the two RCTs was low. Regarding domain,^2^ there were some concerns with one study^14^ as it was conducted using a per‐protocol analysis. All other domains (i.e., missing outcome data, outcome measurements, and selection of reported results) were assigned a low risk of bias. The overall risk of bias in the other study^13^ was low.

Table 3 demonstrates the bias assessment score of the 2 cohort studies.^2^, ^14^ The confounding variables were compared after adjusting for age, which was significantly different in the univariate analysis between groups in one study.^14^ In another study,^2^ TOR was compared using a multilinear regression analysis. Hence, both domains were assigned as low risk of bias. The other domains were also assigned a low risk of bias.

**TABLE 3:** Summary of risk of bias assessment tool for Non‐randomized Studies (RoBANS) assessment in all the cohorts.

	Selection of participants	Selective outcome reporting	Measurement of exposure	Building of outcome assessments	Incomplete outcome data	Overall
Letoumeau 2021	Low	Low	Low	Low	Low	Low
Meirow 2014	Low	Low	Low	Low	Low	Low
Low (%)	100	100	100	100	100	
Some concerns (%)	0	0	0	0	0	
High (%)	0	0	0	0	0	

## 4 SYNTHESIS OF RESULTS

### 4.1 Meta‐analysis of the TOR

**FIGURE 2:**
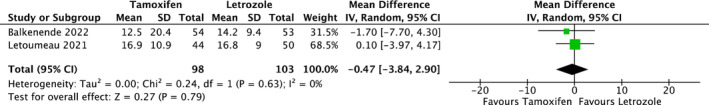
Forest plot of total number of oocyte retrieval data for the comparison between COS with tamoxifen and COS with letrozole; COS, Controlled ovarian stimulation.

Two RCTs provided TOR data for comparison between COS with tamoxifen and COS with letrozole (Figure 2). There was no statistical difference between the two groups (mean Difference, −0.47; 95% CI, [−3.84, 2.90]), and heterogeneity was not observed among studies (*I* ^2^ = 0%, *p* = 0.63).

**FIGURE 3:**
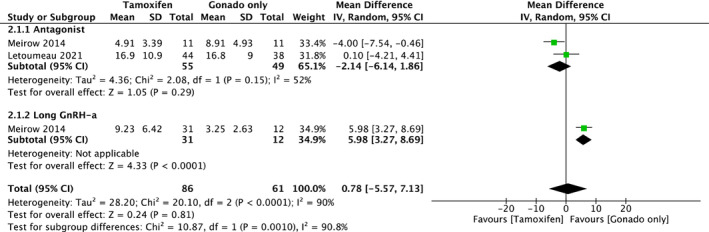
Forest plot of total number of oocyte retrieval data for the comparison between COS with tamoxifen and COS with gonadotropin only; Captions, The outcome is separately shown by type of COS as subgroup analysis. COS, Controlled ovarian stimulation; Gonado, Gonadotropin.

The two cohorts provided TOR data for the comparison between COS with tamoxifen and COS with gonadotropin only. The outcome has been demonstrated according to the type of COS as a subgroup analysis (Figure 3). There was no statistical difference between the two groups in antagonist protocol (mean Difference, −2.14; 95% CI, [−6.14, 1.86]). High heterogeneity was observed among studies (*I* ^2^ = 52%). For the long GnRH‐a protocol, there was only one cohort,^2^ hence, a meta‐analysis could not be conducted.

### 4.2 Meta‐analysis of TMO

**FIGURE 4:**
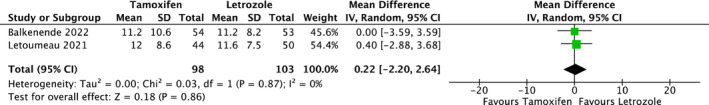
Forest plot of total number of mature oocyte data in comparison COS with Tamoxifen with COS with Letrozole; COS, Controlled ovarian stimulation.

Two RCTs provided TMO data for comparison between COS with tamoxifen and COS with letrozole (Figure 4). There was no statistical difference between the two groups (mean Difference, 0.22; 95% CI, [−2.20, 2.64]). Heterogeneity was not observed among the studies (*I* ^2^ = 0%, *p* = 0.87).

### 4.3 Meta‐analysis of PEL

**FIGURE 5:**
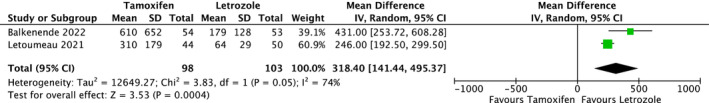
Forest plot of peak estradiol level data for the comparison between COS with tamoxifen and COS with letrozole; Captions, all numbers were shown as actual number divided by 10, to make the forest plot easier to read. COS, Controlled ovarian stimulation.

Two RCTs provided PEL data for the comparison between COS with tamoxifen and COS with letrozole (Figure 5: All numbers were divided by 10 to make the forest plot easier to interpret). COS with letrozole was associated with decreased estrogen levels (mean difference, 3184.4 pg/mL; 95% CI, [1414.4, 4953.7]). Substantial heterogeneity was observed among the studies (*I* ^2^ = 74%, *p* = 0.05).

**FIGURE 6:**
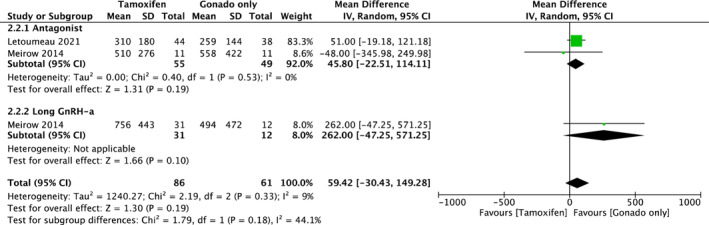
Forest plot of estradiol level data for the comparison between COS with tamoxifen and COS with gonadotropin only; Captions, all numbers were shown as actual number divided by 10, to make the forest plot easier to read. The outcome is separately shown by type of COS as subgroup analysis. COS, Controlled ovarian stimulation; Gonado, Gonadotropin.

In addition, two cohorts provided PEL data for the comparison between COS with tamoxifen and COS with gonadotropin only, and the outcome has been separately demonstrated by the type of COS as a subgroup analysis (Figure 6; all numbers are presented as actual numbers divided by 10 to make the forest plot easier to read). There was no statistical difference between the two groups in antagonist protocol (mean difference, 458.0 pg/mL; 95% CI, [−225.1, 1141.1]). Heterogeneity was not observed among the studies (*I* ^2^ = 0%, *p* = 0.19). In addition to TOR, only one cohort (Meirow 2014) was included in the long GnRH‐a protocol; therefore, we could not conduct a meta‐analysis.

### 4.4 Summary of findings table

Table 4 summarizes the findings. COS with tamoxifen was compared with COS with letrozole in women with breast cancer who underwent fertility preservation.

**TABLE 4:** Summary of findings: COS with tamoxifen compared to COS with letrozole in women with breast cancer who undergo fertility preservation.

Outcomes	Anticipated absolute effects^a^ (95% CI)		Measurement of exposure	Building of outcome assessments^b^
	Risk with COS with Letrozole	Risk with COS with tamoxifen		
Total number of oocytes retrieved	The mean total number of oocytes retrieved was 15.5	MD 0.47 lower (3.84 lower to 2.9 higher)	201 (2 RCTs)	⨁⨁⨁◯ Moderate
Total number of mature oocytes	The mean total number of mature oocytes was 11.4	MD 0.22 higher (2.2 lower to 2.64 higher)	201 (2 RCTs)	⨁⨁⨁◯ Moderate
Peak estradiol level	The mean peak estradiol level was 952.1	MD 3184 higher (1414.4 higher to 4953.7 higher)	201 (2 RCTs)	⨁⨁⨁⨁ High

Abbreviations: CI, confidence interval; COS, controlled ovarian stimulation; MD, mean difference; RCT, Randomized controlled trial.

^a^ The risk in COS with tamoxifen (and its 95% confidence interval) is based on the assumed risk in COS with letrozole and the relative effect of the intervention (and its 95% CI).

^b^ GRADE Working Group grades of evidence; High certainty: we are very confident that the true effect lies close to that of the estimate of the effect; Moderate certainty: we are moderately confident in the effect estimate: the true effect is likely to be close to the estimate of the effect, but there is a possibility that it is substantially different.

The evaluations of certainty of the evidence (GRADE) in TOR and TMO were “moderate” because there was a high imprecision due to the small number of patients included in these studies.

Although the sample size was small, the assessment of GRADE in PEL was deemed “high,” similar to TOR and TMO. This is because the observed disparity was statistically significant, and the magnitude of the difference was substantial.

Table 5 exhibits a summary of the findings in which COS with tamoxifen was compared with COS with gonadotropin only in the antagonist protocol for patients with estrogen‐sensitive breast cancer.

**TABLE 5:** Summary of findings: COS with tamoxifen compared to COS with gonadotropin only for patients with estrogen‐sensitive positive breast cancer.

Outcomes	Anticipated absolute effects^a^ (95% CI)		Measurement of exposure	Building of outcome assessments^b^
	Risk with COS with gonadotropin only	Risk with COS with tamoxifen		
Total number of oocytes retrieved – Antagonist	The mean total number of oocytes retrieved – Antagonist was 15.03	MD 2.14 lower (6.14 lower to 1.86 higher)	104 (2 observational studies)	⨁◯◯◯ Very low
Peak estradiol level – Antagonist	The mean peak estradiol level – Antagonist was 3261	MD 4580 higher (225.1 lower to 1141.1 higher)	104 (2 observational studies)	⨁⨁◯◯ Low

Abbreviations: CI, confidence interval; COS, controlled ovarian stimulation; MD, mean difference.

^a^ The risk in COS with tamoxifen (and its 95% confidence interval) is based on the assumed risk in COS with gonadotropin only and the relative effect of the intervention (and its 95% CI).

^b^ GRADE Working Group grades of evidence; Low certainty: our confidence in the effect estimate is limited: the true effect may be substantially different from the estimate of the effect. Very low certainty: we have very little confidence in the effect estimate: the true effect is likely to be substantially different from the estimate of effect.

The evaluations of GRADE in TOR were “very low” due to severe heterogeneity and small number of included patients. The evaluations of GRADE in PEL were “low,” other than type of COS. All the meta‐analyses included only two eligible studies. Therefore, the sample size was too small to generate funnel plots.

## 5 DISCUSSION

Our meta‐analysis demonstrated no significant difference in TOR and TMO between COS with tamoxifen and COS with letrozole (TOR, 95% CI, [−3.84, 2.90]; TMO, 95% CI, [−2.20, 2.64]). Also, there was no significant difference in TOR between COS with tamoxifen and COS with gonadotropin only in antagonist protocol (TOR, 95% CI, [−6.14, 1.86]). As for PEL, a statistically significant decrease was observed between COS with tamoxifen and COS with letrozole (mean difference, 3184.0, 95% CI, [1414.4, 4953.7]), while the comparison between COS with Tamoxifen and with gonadotropin only in antagonist protocol demonstrated no statistically significant difference in PEL (Mean difference, 458.0; 95% CI, [−225.1, 1141.1]). To the best of our knowledge, this review and meta‐analysis are the first to evaluate the quality of COS with the use of tamoxifen.^7^

There was no difference in TOR and TMO between COS with tamoxifen and COS with letrozole. Oktay, et al. 2005,^5^ whose study was excluded from our screening process, demonstrated no significant difference in TOR and TMO between COS with tamoxifen and COS with letrozole. In addition, the similarity between mature oocyte yields is further supported by a recent European trial of tamoxifen‐gonadotropin versus letrozole‐gonadotropin, which was reported at the 2019 American Society of Reproductive Medicine Conference, but has not yet been published. The findings of all published studies did not demonstrate any significant differences and there was no significant heterogeneity. However, our summary of findings table demonstrates that the evaluations of GRADE were “moderate” in TOR and TMO between COS with tamoxifen and COS with letrozole., because the sample size was too small. Therefore, further studies are warranted. Apart from reduced serum E2 levels, COS with aromatase inhibitors produced little difference in the levels of the other hormones. Yang et al.^15^ reported that high progesterone levels were positively related with the number of mature follicles. In their study, letrozole supplementation did not reduce the incidence of premature progesterone rise/elevation in the late follicular phase. It is imperative to consider other hormones regarding maturation, such as progesterone and FSH, in addition to peak E2 in the future.

A difference in TOR was not observed between COS with tamoxifen and COS with gonadotropin only in the antagonist protocol. Meirow et al.^2^ concluded that there was no overall difference; however, the evaluation of each ovarian stimulation protocol demonstrated significant differences. The heterogeneity of the results was high. Hence, it is difficult to draw conclusions from our review alone. Only one RCT^13^ was conducted that evaluated this outcome. It concluded that there was no significant difference between the two protocols. In the future, further meta‐analyses derived from RCTs will be necessary.

The PEL in COS with tamoxifen was significantly lower than that in COS with letrozole. From a biochemical perspective, a difference between the PEL of the two COS protocols was expected. Tamoxifen increases serum estrogen levels through negative feedback mechanisms that block estrogen receptors,^16^ while letrozole inhibits aromatase activity and suppresses estradiol production.^3^ Although it has been reported that estrogen plays a significant role in the development and growth of BC cells, whether higher serum estradiol levels are related to a high recurrence risk of BC remains controversial.^9^, ^17^ If there is indeed a correlation, it is crucial to elucidate the specific threshold levels of serum estradiol and the impactful exposure durations.^17^ Regarding the risk of high estradiol levels during COS in ART, Arecco et al. demonstrated that patients who underwent COS for fertility preservation had reduced risks of recurrence (RR, 0.58), mortality (RR, 0.54), and event‐free survival (RR, 0.76).^18^ Hence, it is speculated that high estradiol levels induced by COS may not affect the prognosis of BC regardless of treatment timing. However, this systematic review was primarily based on observational studies, necessitating further comprehensive evidence accumulation.

This study had a few limitations. We only assessed TOR, TMO, and PEL as parameters for COS quality; the assessment of the Embryo grade, implantation rate, and other parameters are required for a more comprehensive assessment. Additionally, regarding the pregnancy outcomes, pregnancy rate, live birth rate, and healthy child birthrate were not evaluated due to the limited number of included studies (only 4 studies). As for safety of these protocols, no serious adverse events were reported in the included papers. However, we did not assess the effects of tamoxifen post‐pregnancy, and thus, the disadvantages of discontinuing tamoxifen for maternal prognosis are unclear. For patients who choose to continue tamoxifen during pregnancy, it is noteworthy that there is a reported 12.6% rate of abnormal fetal development, although causality could not be definitively established.^19^

It has been estimated that there will be more high‐quality interventional and observational studies conducted in the future, as the demand for fertility preservation has increased dramatically in the world.^20^ We intend to conduct a review again in the near future as more papers are published, to provide a more accurate and complete picture of the use of tamoxifen for COS in patients with estrogen‐sensitive BC.

In conclusion, the quality of COS did not differ between COS with tamoxifen and COS with letrozole or gonadotropin only. Although the four eligible studies in our review demonstrated a low risk of bias, the sample sizes of these studies were small; therefore, further studies are required.

## CONFLICT OF INTEREST STATEMENT

Tsukasa Yoshida, Osamu Takahashi, Yoko Suzuki, Erika Ota, and Tetsuya Hirata declare that they have no conflicts of interest.

## ETHICS STATEMENT

This review article included no patients, and it did not require approval of Ethics Committee.

## HUMAN RIGHTS STATEMENTS AND INFORMED CONSENT

This study did not contain any human materials.

## ANIMAL STUDIES

Our study is not an animal study.

## CLINICAL TRIAL REGISTRY

Our study is systematic review and meta‐analysis, which did not need clinical trial registry.

## APPENDIX 1 PubMed search strategy

This search was run on 30 October 2022.

1. “Breast Neoplasms”[Mesh] (332,484)
2. “Breast cancer”[Title/Abstract] (321,750)
3. “Receptors, Estrogen”[Mesh] (53,682)
4. “estrogen sensitive”[Title/Abstract] (842)
5. “estrogen receptor positive”[Title/Abstract] (5,551)
6. #1 or #2 (425,284)
7. #3 or #4 or #5 (57,206)
8. Tamoxifen [Title/Abstract] OR Letrozole [Title/Abstract] OR norvadex [Title/Abstract] OR valodex [Title/Abstract] OR femara [Title/Abstract] (27,518)
9. “Aromatase Inhibitors”[Mesh] (6,777)
10. “aromatase inhibitor*”[Title/Abstract] (8,370)
11. #8 or #9 or #10 (33,694)
12. “Ovulation Induction”[Mesh] (14,565)
13. “Gonadotropins”[Mesh] (129,791)
14. “Gonadotropin‐Releasing Hormone”[Mesh] (33,930)
15. “gonadotropin*”[Title/Abstract] (56,773)
16. GnRH [Title/Abstract] (24,140)
17. “controlled ovarian stimulation”[Title/Abstract] (1,672)
18. “Fertility Preservation”[Mesh] (3,618)
19. Fertility [Title/Abstract] OR oncofertility [Title/Abstract] (102,362)
20. “Reproductive Techniques, Assisted”[Mesh] (78,226)
21. “assisted reproductive treatment*”[Title/Abstract] (447)
22. #12 or #13 or #14 or #15 or #16 or #17 or #18 or #19 or #20 or #21 (315,588)
23. #6 and #7 and #11 and # 22 (178)

## APPENDIX 2 Embase search strategy

This search was run on 30 October 2022.

#1 ‘breast cancer’/exp (536525)

#2 ‘breast cancer’:ti, ab, kw (461386)

#3 #1 OR #2 (629383)

#4 ‘estrogen receptor’/exp (104090)

#5 ‘estrogen sensitive’:ti, ab, kw OR ‘estrogen receptor’:ti, ab, kw OR ((estrogen NEAR/1 positive):ti, ab, kw) (70146)

#6 #4 OR #5 (120218)

#7 ‘aromatase inhibitor’/exp (36297)

#8 ‘tamoxifen’/exp (68093)

#9 ‘letrozole’/exp (14174)

#10 ‘aromatase inhibitor’:ti, ab, kw OR tamoxifen:ti, ab, kw OR letrozole:ti, ab, kw OR norvadex:ti, ab, kw OR valodex:ti, ab, kw OR femara:ti, ab, kw (46069)

#11 #7 OR #8 OR #9 OR #10 (94466)

#12 ‘gonadotropin’/exp (33979)

#13 ‘gonadotropin*’:ti, ab, kw OR ‘gnrh’:ti, ab, kw OR ‘ovarian stimulation’:ti, ab, kw (88336)

#14 ‘ovulation induction’/exp (17649)

#15 ‘fertility preservation’/exp (6391)

#16 ‘reproductive procedure’/exp (1781)

#17 ‘embryo’/exp (275035)

#18 ‘cryopreservation’/exp (47413)

#19 ‘oocyte’/exp (87077)

#20 oocyte*:ti, ab, kw OR cryopreservation:ti, ab, kw OR embryo:ti, ab, kw OR ‘reproductive technique*’:ti, ab, kw OR fertility:ti, ab, kw OR oncofertility:ti, ab, kw (348675)

#21 #12 OR #13 OR #14 OR #15 OR #16 OR #17 OR #18 OR #19 OR #20 (662154)

#22 #3 AND #6 AND #11 AND #21 (410)

#23 #22 AND (‘article’/it OR ‘letter’/it OR ‘review’/it OR ‘short survey’/it) (310)

## APPENDIX 3 CENTRAL search strategy

This search was run on 30 October 2022.

#1 (“Breast cancer”):ti, ab, kw (39488)

#2 MeSH descriptor: [Breast Neoplasms] explode all trees (17582)

#3 #1 or #2 (41561)

#4 (Tamoxifen OR Letrozole OR norvadex OR valodex OR femara OR “aromatase inhibitor*”):ti, ab, kw (7940)

#5 MeSH descriptor: [Aromatase Inhibitors] explode all trees (869)

#6 #4 or #5 (8058)

#7 MeSH descriptor: [Gonadotropins] explode all trees (5201)

#8 MeSH descriptor: [Gonadotropin‐Releasing Hormone] explode all trees (2995)

#9 MeSH descriptor: [Ovulation Induction] explode all trees (1678)

#10 MeSH descriptor: [Fertility Preservation] explode all trees (52)

#11 MeSH descriptor: [Reproductive Techniques, Assisted] explode all trees (4051)

#12 (gonadotropin* or GnRH or “ovarian stimulation” or fertility or oncofertility or “assisted reproductive treatment*”):ti, ab, kw (13149)

#13 #7 or #8 or #9 or #10 or #11 or #12 (18122)

#14 #3 and #6 and #13 (273)
